# Risk Factors for Vertebral Compression Fracture Following Spine Stereotactic Body Radiation Therapy

**DOI:** 10.1016/j.adro.2026.102033

**Published:** 2026-03-27

**Authors:** Suchet Taori, Samuel Adida, Shovan Bhatia, James C. Bayley, Pascal O. Zinn, Steven A. Burton, John C. Flickinger, Roberta K. Sefcik, Peter C. Gerszten

**Affiliations:** a School of Medicine, University of Pittsburgh Medical Center, Pittsburgh, Pennsylvania; b Department of Neurological Surgery, University of Pittsburgh Medical Center, Pittsburgh, Pennsylvania; c Department of Radiation Oncology, University of Pittsburgh Medical Center, Pittsburgh, Pennsylvania; d Department of Neurosurgery, Medical University of South Carolina, Charleston, South Carolina

## Abstract

**Purpose:**

Vertebral compression fractures (VCFs) are potential complications following spine stereotactic body radiation therapy (SBRT). However, limited data exist on prognostic factors underlying VCF development following SBRT. The objective of this clinical cohort investigation was to evaluate risk factors for VCF development, VCF type (de novo or progressive), and VCF severity, following SBRT for spinal metastases.

**Methods and Materials:**

A retrospective analysis was performed on a prospectively maintained database of 600 SBRT treatments (779 vertebral segments) for spinal metastases from 2002 to 2024. Exclusion criteria consisted of benign tumors, prior lesion-specific surgical intervention, and <1 month of follow-up. Logistic regression and Fine–Gray subdistribution hazard modeling was performed to evaluate predictors of VCF development.

**Results:**

The median follow-up was 8 months (range, 1-251 months). Fifty-seven (10%) VCFs were identified following SBRT: 29 (5%) de novo VCFs and 28 (5%) progressive VCFs. The median time-to-VCF was 6 months (range, 1-83 months). The 1- and 3-year cumulative incidence of VCF was 11% (95% CI, 8.1%-15%) and 21% (95% CI, 15%-27%), respectively. On multivariable analysis, female sex (odds ratio [OR], 1.06; 95% CI, 1.02-1.11; *P* = .007), lumbar lesions (OR, 1.05; 95% CI, 1.00-1.11; *P* = .049), and pre-existing VCF (OR, 1.01; 95% CI, 1.00-1.02; *P* = .009) were significantly associated with any VCF development, whereas a greater Spinal Instability Neoplastic Scores (SINS) at SBRT trended toward significance (OR, 1.07; 95% CI, 1.00-1.14; *P* = .060). Osteolytic disease (OR, 1.04; 95% CI, 1.00-1.07; *P* = .042) and epidural tumor extension (OR, 1.04; 95% CI, 1.01-1.07; *P* = .022) were associated with de novo VCF development, whereas only pre-existing VCF (OR, 1.08; 95% CI, 1.04-1.12; *P* < .001) was significantly associated with progressive VCF development. Thirty-three (58%) VCFs required subsequent surgical stabilization. On multivariable analysis, lumbar lesions (OR, 1.07; 95% CI, 1.02-1.12; *P* = .004), pre-existing VCF (OR, 1.07; 95% CI, 1.01-1.13; *P* = .026), and a greater SINS at SBRT (OR, 1.02; 95% CI, 1.01-1.02; *P* = .002) were significantly associated with VCFs requiring stabilization.

**Conclusions:**

This large clinical cohort investigation successfully identified patient subgroups with increased risks of developing VCFs following spine SBRT for metastatic disease. Identifying these at-risk subpopulations may guide additional follow-up surveillance and consideration of individualized discussions regarding potential conservative or stabilization management approaches, particularly for those patients with multiple underlying risk factors.

## Introduction

In recent years, randomized controlled trials have demonstrated spine stereotactic body radiation therapy (SBRT) to be a minimally invasive, safe, and effective treatment modality for the management of vertebral spinal metastases.^1^, ^2^, ^3^, ^4^ SBRT delivers precise high-dose radiation to the tumor site and offers high rates of local control, adequate patient pain relief, and limited adverse radiation effects (AREs).^5^, ^6^, ^7^, ^8^, ^9^ AREs following SBRT include fatigue, nausea, dermatitis, esophagitis, pneumonitis, pain flare, radiculopathy, myelopathy, and vertebral compression fractures (VCFs).^10^, ^11^, ^12^, ^13^, ^14^, ^15^ Although most toxicities are low-grade and self-limiting, myelopathy and VCFs are 2 delayed consequences of SBRT that warrant careful consideration during SBRT dose planning and follow-up.^13^^,^^14^^,^^16^, ^17^, ^18^, ^19^ SBRT-induced myelopathy is a potentially serious ARE, but it is rare, and risks can be minimized by reducing the maximal spinal cord dose.^14^^,^^16^^,^^20^ VCFs following SBRT are more common, ranging from 5% to 39% in the literature, and can lead to debilitating symptoms such as progressive pain and neurologic deficit.^21^, ^22^, ^23^, ^24^, ^25^, ^26^ Despite their relatively high incidence, clinical and radiographic predictors of VCF development vary considerably, owing to patient heterogeneity and differing SBRT treatment parameters between studies.^21^, ^22^, ^23^^,^^26^, ^27^, ^28^, ^29^, ^30^ Further, few studies report specifically on predictors of VCF severity.^26^^,^^27^^,^^31^^,^^32^

The purpose of this large, single-institution clinical cohort investigation was to identify significant prognostic factors associated with the development of VCFs following SBRT, including the timing of their occurrence and the likelihood of requiring stabilization. As systemic therapies continue to improve and patients with metastatic spinal disease live longer, identification of populations that are most susceptible to late toxicities following SBRT will better inform clinical decision-making and patient management.^33^^,^^34^

## Methods and Materials

### Study cohort

A retrospective review of a prospectively maintained database for patients with metastatic spinal tumors treated with SBRT at the [University of Pittsburgh Medical Center] between 2002 and 2024 was conducted. This study was approved by the [University of Pittsburgh] Human Research Protection Office (#PRO08120394) and received institutional review board approval. All patients consented to the SBRT procedure. Inclusion criteria were defined as the following: (1) patients with metastatic spinal tumors diagnosed radiographically by magnetic resonance imaging, histopathologically by stereotactic biopsy, and/or documented medical history; (2) complete radiographic imaging and clinical data; and (3) sufficient follow-up data. Lesions were excluded if they: (1) were benign or (2) had undergone prior lesion-specific spine surgery, including but not limited to separation surgery, posterior laminectomy, corpectomy, vertebral body replacement, costotransversectomy, total en bloc spondylectomy, vertebroplasty, and kyphoplasty. Treatments in which tumors radiographically progressed following SBRT and before or concurrent with VCF development were not recorded as VCF events in the analysis. This was done to differentiate VCFs caused by tumor progression from those intrinsic to the SBRT treatment. SBRT treatments involving contiguous multilevel vertebral segments were denoted as single treatments for analysis. This permitted tumor and SBRT dosimetry parameters to evaluate the complete disease process, as opposed to individual effects on multiple involved vertebral segments. The inclusion, exclusion criteria, and analysis procedures in this study follow those of similar prior reports.^21^^,^^23^^,^^24^^,^^26^^,^^28^^,^^30^

Patient demographic data, prior treatment history, SBRT clinical and radiographic treatment parameters, and SBRT dosimetry data were recorded. The epidural spinal cord compression (ESCC) scale was used to evaluate epidural tumor involvement.^35^ Spinal Instability Neoplastic Scores (SINS) were calculated using a validated scoring system for every tumor to assess spinal instability and were stratified as stable (0-6 points), potentially unstable (7-12 points), and unstable (13-18 points).^36^ SINS is a composite metric derived from 6 component domains, each potentially reflecting a distinct biomechanical contributor to spinal stability. Tumor location may reflect regional biomechanical loading differences along the spinal column; mechanical pain may reflect motion-dependent load transfer; baseline vertebral body collapse may reflect pre-existing structural compromise; radiographic misalignment may reflect altered load transmission and segmental instability; lytic bone quality may reflect reduced load-bearing capacity; and posterolateral involvement may reflect compromise of stabilizing posterior elements. These features may contribute to VCF risk through mechanisms that are not fully captured by the aggregate SINS score alone. As such, SINS components were analyzed individually to determine whether specific biomechanically-relevant instability features retained independent prognostic significance beyond the overall SINS score.

### Study outcomes and follow-up

The primary outcome of this study was the development of VCFs following SBRT. Prognostic factors were analyzed to evaluate their association with the risk of any VCF development, time-to-VCF development, de novo and progressive VCF development, and the likelihood of a VCF requiring surgical intervention. VCF development was assessed through a retrospective review of neuroradiology imaging reports and by comparison of pre-SBRT and post-SBRT imaging. Imaging reports were documented and assessed primarily by the same team of neuroradiologists over the study period and discrepancies between reports were jointly evaluated by the team of neuroradiologists at our institution. Time-to-VCF development was calculated as the time between SBRT and VCF development or the last follow-up. De novo VCFs were defined as new vertebral fractures or collapse deformity at the treated level following SBRT. Progressive VCFs were defined as fractures that were present before SBRT and progressed at the same vertebral level following treatment. Tumor progression was defined as a clear increase in tumor volume or linear dimension, new or progressive epidural disease, or neurologic decline linked to epidural disease with corresponding magnetic resonance imaging changes. These definitions are consistent with the prior literature.^21^^,^^24^^,^^26^^,^^28^^,^^37^ Patients were followed up at 1 month following SBRT, then in 3-month intervals for 1 year, and annually thereafter.

### SBRT technique

The SBRT technique used in this study has previously been described in detail.^38^, ^39^, ^40^ Treatment plans were tailored for each patient, considering tumor size, geometry, and proximity to critical structures such as the spinal cord and neurovascular tissues. Treatment planning was performed using the Accuray (Accuray), Pinnacle (Philips), or Eclipse (Varian) treatment planning systems. The spinal cord was contoured from the foramen magnum to the level of the conus medullaris, and the cauda equina was contoured from the conus medullaris inferiorly to the level of the sacrum. SBRT was delivered with the CyberKnife Robotic Radiosurgery System (Accuray), Synergy Radiosurgery System (Elekta), and the TrueBeam Radiotherapy System (Varian Medical Systems). Dose constraints for the spinal cord, cauda equina, and organs-at-risk were optimized on a case-by-case basis and followed institutional and published international consensus guidelines at the time of treatment.^41^ A multidisciplinary team comprising neurosurgeons, radiation oncologists, and medical physicists reviewed and validated each treatment plan before administration. Biologically effective [prescription] doses (BED_2_ and BED_10_), used to quantify and compare the biological effect of radiation on the spinal cord and treated tumors across fractionation schemes, were approximated using an α/β ratio of 2 and 10, respectively.^42^^,^^43^ BED_2_ was used to approximate late-responding normal tissue sensitivity and to standardize spinal cord/cauda equina dose constraints across fractionation schedules. BED_10_ was used to characterize tumor/early-responding tissue dose intensity across SBRT regimens. Because the radiobiologic α/β ratio of tumor-associated vertebral bone is variable and VCF risk likely reflects a continuum of effects spanning tumor irradiation and adjacent normal tissue exposure, documenting both BED_2_ and BED_10_ allows evaluation of SBRT dose intensity across this spectrum.

### Statistical analysis

Statistical analysis was performed using GraphPad Prism Version 9 (GraphPad). Cumulative incidence curves, with death as a competing risk, were created to depict temporal VCF development. Fine–Gray subdistribution univariable and multivariable hazard model analysis, using death as a competing factor, was conducted to assess the temporal relationship between prognostic factors and VCF development (RStudio). Univariable and multivariable logistic regression modeling was performed to identify risk factors associated with VCF development, type (de novo or progressive), and the likelihood of subsequent surgical stabilization (RStudio). Hazard ratios (HRs) with 95% confidence intervals (CIs) were calculated to assess time-dependent prognostic factor significance in Fine–Gray models and odds ratios (ORs) with 95% CIs were calculated to assess cross-sectional risk prognostic factor significance in the logistic regression models. To ensure predictive independence, only factors significant in univariable analysis were included in the multivariable analysis. A 2-tailed *P* < .05 was considered statistically significant.

## Results

### Patient demographics

A total of 404 patients (52% male) with 600 lesions involving 779 vertebral bodies met the inclusion criteria and were included in this study. The median age at SBRT was 60 years (range, 22-94 years), and the median KPS at SBRT was 80% (range, 50-100). Primary tumor histology per treatment consisted of breast (134 lesions, 22%), lung (113 lesions, 19%), renal (97 lesions, 16%), gastrointestinal (73 lesions, 12%), melanoma (38 lesions, 6%), prostate (32 lesions, 5%), thyroid (26 lesions, 4%), and other rarer (87 lesions, 15%) types. Three-hundred ninety-seven (66%) treatments involved vertebral segments previously irradiated with external beam radiation therapy (EBRT), and 421 (70%) treatments involved patients treated with cytotoxic chemotherapy for their systemic disease. The degree of systemic disease per treatment was solitary (108 lesions, 18%), oligometastatic (332 lesions, 55%), polymetastatic (123 lesions, 21%), or unknown at the time of this study (37 lesions, 6%). Patient demographics are reported in Table E1.

### SBRT characteristics

The 600 SBRT treatments were for tumors in the cervical (81 treatments, 14%), thoracic (266 treatments, 44%), lumbar (164, 27%), and sacral (89 treatments, 15%) spine. Two-hundred ninety-six (49%) treatments were for lesions without epidural extension (ESCC score of 0). ESCC scores for the remaining treatments were 1a (92 treatments, 15%), 1b (82 treatments, 14%), 1c (54 treatments, 9%), 2 (58 treatments, 10%), and 3 (18 treatments, 3%). Two-hundred fifteen (36%) treatments involved paraspinal musculature, and 370 (62%) involved posterolateral involvement of spinal elements. Bone quality included lytic (395 treatments, 60%), blastic (92 treatments, 15%), and mixed (149 treatments, 25%). One-hundred sixty-two (27%) treatments involved baseline vertebral body collapse. Radiographic spinal misalignment was observed in 99 (17%) cases. Using the SINS criteria, tumors per treatment were considered stable (0-6; 249 treatments, 42%), potentially unstable (7-12; 303 treatments, 51%), and unstable (13-18; 48 treatments, 8%).

Most treatments (493 treatments, 82%) were performed in a single fraction. For single-fraction SBRT, the median tumor volume was 30.4 cm^3^ (range, 0.1-232 cm^3^), with a median margin dose of 16 Gy (range, 8-20 Gy) and a median maximum dose of 20 Gy (range, 13-32 Gy). One-hundred seven (18%) treatments were performed in multiple fractions. Fraction regimens included 2 fractions (21 treatments, 4%), 3 fractions (73 treatments, 12%), and 5 fractions (13 treatments, 2%). The median tumor volume for multifraction treatments was 32.1 cm^3^ (range, 2.1-264.3 cm^3^), with a median margin dose of 24 Gy (range, 18-35 Gy) and a median maximum dose of 45 Gy (range, 29-60 Gy). The median isodose line for all treatments was 80% (range, 37-100). For the entire cohort, the BED_2_ delivered to the spinal cord or cauda equina was 144 Gy (range, 40-220 Gy). Full radiographic and SBRT dosimetry parameters are reported in Table E2.

### VCF development

**Figure 1:**
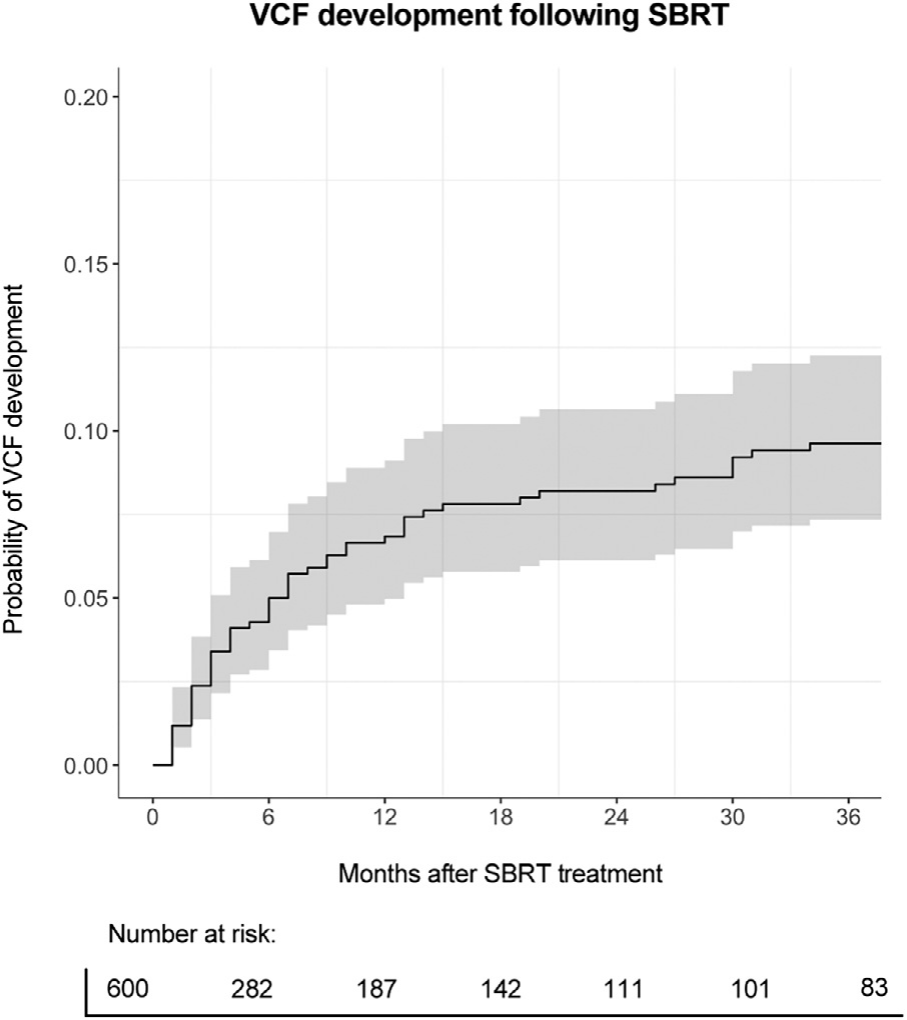
Cumulative incidence risk of VCF development following SBRT. *Abbreviations:* SBRT = stereotactic body radiation therapy; VCF = vertebral compression fracture.

The median follow-up after SBRT was 8 months (range, 1-251 months). There were 57 (10%) instances of VCFs following SBRT. Twenty-nine (5%) VCFs were progressive, and 28 (5%) VCFs were de novo. The median time-to-VCF development was 6 months (range, 1-83 months). After diagnosis, VCFs were managed either conservatively (24 VCFs, 42%) or operatively with percutaneous cement augmentation (25 VCFs, 44%) and open spinal reconstruction surgery (8 VCFs, 14%). Cumulative incidence rates of VCFs at 6 months, 1 year, and 3 years following SBRT were 7.3% (95% CI, 5%-10%), 11% (95% CI, 8.1%-15%), and 21% (95% CI, 15%-27%), respectively (Fig. 1). Descriptive statistics regarding VCF development are reported in Table 1.

**Table 1:** Development of vertebral compression fractures following spine SBRT

Characteristics	Value
Median follow-up (mo)	8 (1-251)
VCF development following SBRT (no.)	57 (10%)
De novo VCFs	29 (5%)
Progressive VCFs	28 (5%)
Time-to-VCF development (mo)	6 (1-83)
Cumulative incidence of VCFs at:	
6 mo	7.3% (95% CI, 5%-10%)
1 y	11% (95% CI, 8.1%-15%)
3 y	21% (95% CI, 15%-27%)
Management of VCFs (no. of VCFs/total no. of VCFs)	
Conservative	24 (42%)
Percutaneous cement augmentation	25 (44%)
Open spinal reconstruction surgery	8 (14%)

*Abbreviations:* SBRT = stereotactic body radiation therapy; VCF = vertebral compression fracture.

Values are reported as number (percentage) or median (range). All % are divided by the total number of SBRT treatments unless otherwise stated.

### VCF prognostic factor analysis

Predictive risk factors were first analyzed for any VCF development following SBRT. Univariable logistic regression analysis identified several significant predictors of VCF development: female sex (OR, 1.06; 95% CI, 1.01-1.11; *P* = .017), lumbar lesions (OR, 1.05; 95% CI, 1.00-1.12; *P* = .047), pre-existing VCF at SBRT (OR, 1.06; 95% CI, 1.01-1.13; *P* = .020), and a greater SINS at SBRT (OR, 1.08; 95% CI, 1.00-1.16; *P* = .041). On multivariable logistic regression analysis, female sex (OR, 1.06; 95% CI, 1.02-1.11; *P* = .007), lumbar lesions (OR, 1.05; 95% CI, 1.00-1.11; *P* = .049), and pre-existing VCF at SBRT (OR, 1.01; 95% CI, 1.01-1.02; *P* = .009) remained significant predictors of any VCF development following SBRT, whereas a greater SINS at SBRT trended toward significance (OR, 1.07; 95% CI, 1.00-1.14; *P* = .060) (Table 2).

**Table 2:** Prognostic risk factors associated with the development of any VCF following spine SBRT using logistic regression and Fine–Gray subdistribution hazard modeling

	Univariable			Multivariable		
Logistic regression	OR	95% CI	*P*	OR	95% CI	*P*
Female sex	1.06	(1.01-1.11)	**.017**	1.06	(1.02-1.11)	**.007**
Age (continuous)	1.00	(1.00-1.00)	.2			
KPS > 80	1.01	(0.95-1.06)	.8			
Oligo- or polymetastatic disease	0.95	(0.90-1.01)	.087			
Breast histology	1.02	(0.97-1.07)	.4			
Prior chemotherapy	1.06	(1.00-1.13)	.060			
Prior EBRT	0.99	(0.95-1.04)	.8			
Lumbar lesion	1.05	(1.00-1.12)	**.047**	1.05	(1.00-1.11)	**.049**
Tumor volume (continuous)	1.00	(0.99-1.01)	.7			
>1 VB irradiated in one treatment	1.05	(1.00-1.12)	.067			
BED_2_ (continuous)	0.99	(0.98-1.00)	.12			
BED_10_ (continuous)	0.97	(0.94-1.01)	.10			
Single-fraction SBRT	0.99	(0.93-1.05)	.7			
Paraspinal musculature extension at SBRT	1.02	(0.97-1.07)	.5			
ESCC > 0 at SBRT	1.04	(0.99-1.09)	.091			
Junctional location at SBRT	0.96	(0.91-1.00)	.074			
Baseline pain at SBRT	1.06	(0.92-1.14)	.14			
Lytic lesion	0.96	(0.92-1.01)	.2			
Radiographic spinal misalignment at SBRT	1.04	(0.98-1.12)	.2			
Posterolateral spine involvement at SBRT	1.03	(0.99-1.08)	.2			
Pre-existing VCF at SBRT	1.19	(1.13-0.22)	**<.001**	1.01	(1.00-1.02)	**.009**
SINS at SBRT (continuous)	1.08	(1.00-1.16)	**.041**	1.07	(1.00-1.14)	.060

	Univariable			Multivariable		
Fine–Gray	HR	95% CI	*P*	HR	95% CI	*P*
Female sex	1.86	(1.08-3.20)	**.024**	1.76	(1.08-2.87)	**.023**
Age > 60 y	1.08	(0.64-1.80)	.8			
Age (continuous)	0.99	(0.97-1.00)	.2			
KPS > 80	1.06	(0.58-1.93)	.9			
Oligo- or polymetastatic disease	0.62	(0.35-1.11)	.11			
Breast histology	1.15	(0.71-1.86)	.57			
Prior chemotherapy	2.10	(0.90-4.32)	.087			
Prior EBRT	0.95	(0.55-1.65)	.9			
Lumbar lesion	1.85	(1.09-3.16)	**.024**	1.79	(1.12-2.87)	**.015**
Tumor volume > 20 cc	1.17	(0.69-1.97)	.6			
Tumor volume (continuous)	1.00	(0.99-1.00)	.7			
>1 VB irradiated in one treatment	1.73	(1.00-3.00)	**.049**	1.83	(0.88-3.81)	.10
BED_2_ > 150 Gy	1.10	(0.58-2.08)	.8			
BED_2_ (continuous)	0.99	(0.99-1.00)	.10			
BED_10_ > 40 Gy	0.63	(0.39-1.02)	.060			
BED_10_ (continuous)	0.98	(0.95-1.01)	.12			
Single-fraction SBRT	1.03	(0.51-2.10)	.9			
Paraspinal musculature extension at SBRT	1.21	(0.72-2.06)	.5			
ESCC > 0 at SBRT	1.65	(0.97-2.81)	.063			
Junctional location at SBRT	0.61	(0.35-1.07)	.083			
Baseline pain at SBRT	2.33	(0.74-7.37)	.15			
Lytic lesion	0.70	(0.42-1.17)	.2			
Radiographic spinal misalignment at SBRT	1.58	(0.85-2.93)	.2			
Posterolateral spine involvement at SBRT	1.56	(0.88-2.74)	.12			
Pre-existing VCF at SBRT	3.04	(1.54-5.99)	**.001**	1.75	(1.12-2.74)	**.014**
SINS at SBRT (continuous)	1.08	(1.02-1.15)	**.011**	1.10	(1.02-1.19)	**.017**

*Abbreviations:* BED_2_ = biologically equivalent prescription dose with α/β = 2 Gy; BED_10_ = biologically equivalent prescription dose with α/β = 10 Gy; ESCC = epidural spinal cord compression score; HR = hazard ratio; KPS = Karnofsky performance score; OR = odds ratio; RT = radiation therapy; SBRT = stereotactic body radiation therapy; SINS = spinal instability neoplastic score; VB = vertebral body; VCF = vertebral compression fracture.

**Figure 2:**
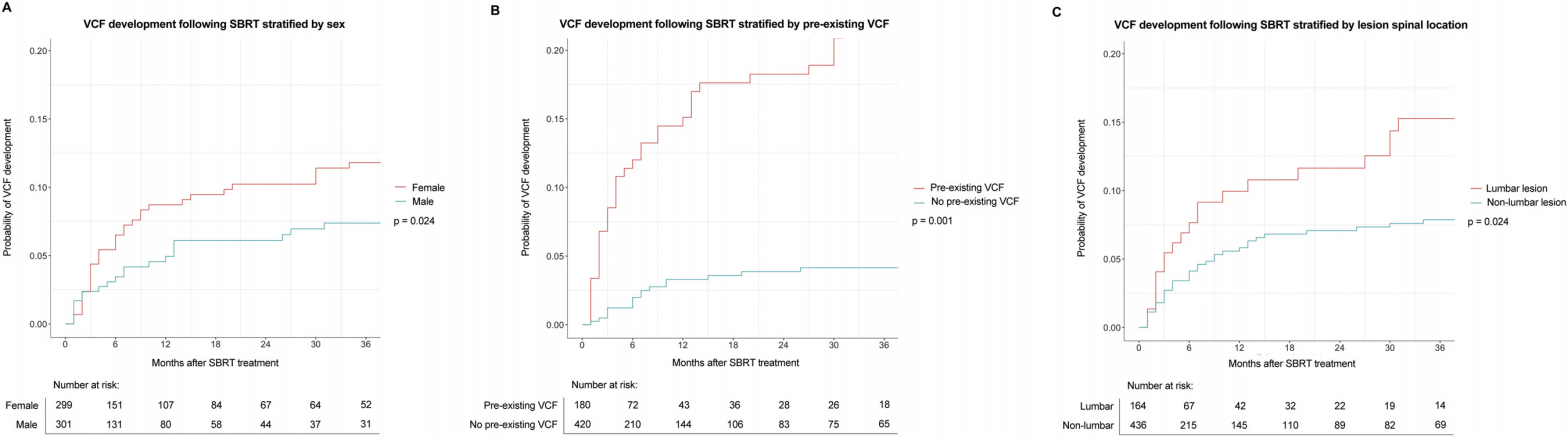
**(**A) Cumulative incidence risk of VCF development following SBRT stratified by sex (male or female). (B) Cumulative incidence risk of VCF development following SBRT stratified by pre-existing VCF. (C) Cumulative incidence risk of VCF development following SBRT stratified by lesion location. *Abbreviations:* SBRT = stereotactic body radiation therapy; VCF = vertebral compression fracture.

A complimentary Fine–Gray competing-risk regression model was performed to evaluate temporal predictors of VCF development, in which death was designated as a competing event **(**Table 2). Concordant with the logistic regression model, univariable analysis revealed that female sex (HR, 1.86; 95% CI, 1.08-3.20; *P* = .024), lumbar lesions (HR, 1.85; 95% CI, 1.09-3.16; *P* = .024), more than 1 vertebral level irradiated in 1 treatment (HR, 1.73; 95% CI, 1.00-3.00; *P* = .049), pre-existing VCF at SBRT (HR, 3.04; 95% CI, 1.54-5.99; *P* = .001), and a greater SINS at SBRT (HR, 1.08; 95% CI, 1.02-1.15; *P* = .011) were significant predictors of any VCF development following SBRT. On multivariable analysis, female sex (HR, 1.76; 95% CI, 1.08-2.87; *P* = .023), lumbar lesions (HR, 1.79; 95% CI, 1.12-2.87; *P* = .015), pre-existing VCF at SBRT (HR, 1.75; 95% CI, 1.12-2.74; *P* = .014), and a greater SINS at SBRT (HR, 1.10; 95% CI, 1.02-1.19; *P* = .017) remained significant predictors of any VCF development following SBRT (Fig. 2A, B).

Next, prognostic risk factors associated with de novo (Table 3) and progressive (Table 3) VCF development were examined. Univariable logistic regression revealed that female sex (OR, 1.04; 95% CI, 1.00-1.07; *P* = .035), epidural extension (ESCC > 0) at SBRT (OR, 1.04; 95% CI, 1.00-1.07; *P* = .044), and lytic lesions (OR, 1.04; 95% CI, 1.00-1.07; *P* = .039) were associated with de novo VCF development. On multivariable analysis, epidural extension (ESCC > 0) at SBRT (OR, 1.04; 95% CI, 1.01-1.07; *P* = .022) and lytic lesions (OR, 1.04; 95% CI, 1.00-1.07; *P* = .042) remained significantly associated with de novo VCF development. The only significant predictive factor of progressive VCF development was pre-existing VCF at SBRT (OR, 1.08; 95% CI, 1.04-1.12; *P* < .001).

**Table 3:** Prognostic risk factors associated with *de novo* and progressive VCFs following spine SBRT

		Univariable						Multivariable				
De novo VCF development		OR		95% CI		*P*		OR	95% CI		*P*	
Female sex		1.04		(1.00-1.07)		**.035**		1.03	(1.00-1.07)		.056	
Age (continuous)		0.97		(0.94-1.00)		.059						
KPS > 80		1.03		(0.99-1.07)		.14						
Oligo- or polymetastatic disease		0.98		(0.94-1.02)		.4						
Breast histology		1.03		(0.99-1.06)		.2						
Prior chemotherapy		1.03		(0.98-1.07)		.2						
Prior EBRT		1.00		(0.96-1.04)		>.9						
Lumbar lesion		1.02		(0.98-1.06)		.3						
Tumor volume (continuous)		0.99		(0.98-1.01)		.14						
>1 VB irradiated in 1 treatment		1.03		(0.99-1.07)		.2						
BED_2_ (continuous)		1.00		(0.99-1.00)		.9						
BED_10_ (continuous)		0.99		(0.95-1.04)		.6						
Single-fraction SBRT		0.99		(0.94-1.03)		0.6						
Paraspinal musculature extension at SBRT		1.01		(0.98-1.05)		.5						
ESCC > 0 at SBRT		1.04		(1.00-1.07)		**.044**		1.04	(1.01-1.07)		**.022**	
Junctional location at SBRT		1.00		(0.97-1.04)		.8						
Baseline pain at SBRT		1.02		(0.97-1.08)		.5						
Lytic lesion		1.04		(1.00-1.07)		**.039**		1.04	(1.00-1.07)		**.042**	
Radiographic spinal misalignment at SBRT		1.03		(0.98-1.07)		.3						
Posterolateral spine involvement at SBRT		1.01		(0.97-1.04)		.7						
SINS at SBRT (continuous)		1.09		(0.99-1.21)		.085						

			Univariable					Multivariable				
Progressive VCF development			OR		95% CI		*P*	OR		95% CI		*P*
Female sex			1.02		(0.99-1.05)		.2					
Age (continuous)			1.00		(0.99-1.00)		.2					
KPS > 80			0.98		(0.94-1.02)		.3					
Oligo- or polymetastatic disease			0.97		(0.92-1.01)		.14					
Breast histology			1.00		(0.96-1.03)		.8					
Prior chemotherapy			1.03		(0.98-1.07)		.2					
Prior EBRT			1.00		(0.97-1.03)		.8					
Lumbar lesion			1.03		(0.99-1.07)		.10					
Tumor volume (continuous)												
>1 VB irradiated in one treatment			1.02		(0.98-1.06)		.3					
BED_2_ (continuous)			1.00		(0.99-1.00)		.9					
BED_10_ (continuous)			0.96		(0.92-1.00)		.073					
Single-fraction SBRT			1.00		(0.96-1.04)		>.9					
Paraspinal musculature extension at SBRT			1.01		(0.97-1.04)		.7					
ESCC > 0 at SBRT			1.01		(0.97-1.04)		.8					
Junctional location at SBRT			0.98		(0.94-1.02)		.4					
Baseline pain at SBRT			1.04		(0.98-1.09)		.2					
Lytic lesion			1.00		(0.97-1.04)		>.9					
Radiographic spinal misalignment at SBRT			1.02		(0.97-1.06)		.5					
Posterolateral spine involvement at SBRT			1.03		(0.99-1.06)		.14					
Pre-existing VCF at SBRT			1.17		(1.13-1.20)		**<.001**	1.08		(1.04-1.12)		**<.001**
SINS at SBRT (continuous)			1.06		(0.95-1.17)		.3					

*Abbreviations:* BED_2_ = biologically equivalent prescription dose with α/β = 2 Gy; BED_10_ = biologically equivalent prescription dose with α/β = 10 Gy; ESCC = epidural spinal cord compression score; KPS = Karnofsky performance score; OR = odds ratio; RT = radiation therapy; SBRT = stereotactic body radiation therapy; SINS = spinal instability neoplastic score; VB = vertebral body; VCF = vertebral compression fracture.

Lastly, risk factors associated with VCFs requiring subsequent stabilization surgery were identified (Table 4). On univariable logistic regression analysis, lumbar lesions (OR, 1.06; 95% CI, 1.01-1.12; *P* = .018), >1 vertebral level irradiated in a single treatment (OR, 1.05; 95% CI, 1.00-1.11; *P* = .037), tumor epidural extension (ESCC > 0) at SBRT (OR, 1.06; 95% CI, 1.02-1.11; *P* = .006), baseline pain at SBRT (OR, 1.08; 95% CI, 1.02-0.16; *P* = .015), radiographic spinal misalignment at SBRT (OR, 1.11; 95% CI, 1.05-1.17; *P* < .001), pre-existing VCF at SBRT (OR, 1.05; 95% CI, 1.01-1.11; *P* = .017), and a greater SINS at SBRT (OR, 1.01; 95% CI, 1.01-1.02; *P* < .001) were significant predictors of VCFs requiring subsequent stabilization surgery. On multivariable analysis, only lumbar lesions (OR, 1.07; 95% CI, 1.02-1.12; *P* = .004), pre-existing VCF at SBRT (OR, 1.07; 95% CI, 1.01-1.13; *P* = .026), and a greater SINS at SBRT (OR, 1.02; 95% CI, 1.01-1.02; *P* = .002) were significantly associated with subsequent VCF development requiring stabilization surgery.

**Table 4:** Prognostic risk factors associated with the development of VCFs requiring subsequent spinal stabilization following spine SBRT

	Univariable			Multivariable		
	OR	95% CI	*P*	OR	95% CI	*P*
Female sex	1.02	(0.98-1.06)	.3			
Age (continuous)	1.00	(1.00-1.00)	.8			
KPS > 80	1.04	(0.99-1.09)	.2			
Oligo- or polymetastatic disease	0.99	(0.93-1.04)	.7			
Breast histology	1.01	(0.96-1.05)	.8			
Prior chemotherapy	0.98	(0.93-1.03)	.4			
Prior EBRT	1.01	(0.96-1.05)	.8			
Lumbar lesion	1.06	(1.01-1.12)	**.018**	1.07	(1.02-1.12)	**.004**
Tumor volume (continuous)	1.00	(0.99-1.01)	.7			
>1 VB irradiated in one treatment	1.05	(1.00-1.11)	**.037**	1.03	(0.99-1.07)	.12
BED_2_ (continuous)	1.00	(0.99-1.01)	.7			
BED_10_ (continuous)	1.00	(0.97-1.04)	.6			
Single-fraction SBRT	1.00	(0.95-1.06)	>.9			
Paraspinal musculature extension at SBRT	1.01	(0.97-1.05)	.7			
ESCC > 0 at SBRT	1.06	(1.02-1.11)	**.006**	1.01	(0.96-1.06)	.5
Junctional location at SBRT	1.00	(0.96-1.04)	>.9			
Baseline pain at SBRT	1.08	(1.02-1.16)	**.015**	1.05	(0.98-1.12)	.15
Lytic lesion	1.00	(0.96-1.05)	.8			
Radiographic spinal misalignment at SBRT	1.12	(1.05-1.17)	**<.001**	1.04	(0.98-1.11)	.2
Posterolateral spine involvement at SBRT	1.03	(0.98-1.07)	.2			
Pre-existing VCF at SBRT	1.11	(1.06-1.16)	**<.001**	1.07	(1.01-1.13)	**.026**
SINS at SBRT (continuous)	1.01	(1.01-1.02)	**<.001**	1.02	(1.01-1.02)	**.002**

*Abbreviations:* BED_2_ = biologically equivalent prescription dose with α/β = 2 Gy; BED_10_ = biologically equivalent prescription dose with α/β = 10 Gy; ESCC = epidural spinal cord compression score; KPS = Karnofsky performance score; OR = odds ratio; RT = radiation therapy; SBRT = stereotactic body radiation therapy; SINS = spinal instability neoplastic score;. VB = vertebral body;. VCF = vertebral compression fracture.

## Discussion

This clinical cohort investigation was performed to evaluate a large single-institution experience of VCF development following SBRT for malignant spine tumors. Significant prognostic factors associated with VCF development after SBRT were identified, including subanalyses on select VCF patient cohorts.

### VCF development following SBRT

Rates of VCF development following SBRT range from 5% to 40% in the literature, with de novo VCF and progressive VCF rates ranging from 3% to 20%, and 3% to 15%, respectively.^21^, ^22^, ^23^, ^24^^,^^26^^,^^28^^,^^30^^,^^32^^,^^44^^,^^45^ In the present study, the crude VCF development rate was 10%, with 5% being de novo VCFs and 5% being progressive VCFs. Although these rates fall within ranges from the literature, extending the median follow-up time of 8 months in this study (compared to 7-21 months in prior reports) may have captured additional slow-forming VCFs. This is further evident as the median time-to-VCF development in the literature ranges from 2 to 13 months, excluding 1 report from Rose et al.^27^ In this study, 58% of VCFs were managed with subsequent stabilization surgery; rates from prior reports range from 25% to 60%.^21^, ^22^, ^23^, ^24^^,^^26^^,^^28^^,^^30^^,^^32^^,^^44^^,^^45^ To better contextualize this study with other similar published reports, a comparison can be visualized inTable E3.

In the present study, female sex, pre-existing VCF at SBRT, and lumbar lesions were identified as significant predictors of any VCF development following SBRT using multivariable logistic regression modeling while a greater SINS at SBRT trended toward significance. A complementary Fine–Gray analysis was used to confirm these multivariable findings with the only difference being statistical significance when assessing a greater SINS at SBRT as a risk factor for VCF development. The rationale for employing both models was to strengthen the robustness and interpretability of the results. The rationale for employing both models was to strengthen the robustness and interpretability of the results. Logistic regression was used to assess cross-sectional associations between prognostic factors and the occurrence of VCF (binary outcome: yes/no) over the follow-up period, reflecting overall fracture susceptibility. Because patients with metastatic disease may die before developing a VCF and follow-up duration was heterogeneous, Fine–Gray competing-risk regression was additionally performed to model time-to-VCF while accounting for death as a competing event. These approaches address complementary clinical questions: logistic regression estimates the association between predictors and the likelihood of VCF occurrence, whereas Fine–Gray regression quantifies the association between predictors and the cumulative incidence of VCF over time in the presence of competing mortality. Presenting both models allows risk interpretation in both a binary and time-dependent competing-risk framework, which is particularly relevant in cohorts such as these with heterogeneous competing mortality.

Although not a commonly reported predictor of VCF development following SBRT, prior reports have found the incidence of VCFs at baseline to be greater in women than in men.^46^^,^^47^ Additionally, postmenopausal and elderly women, characteristic of women in this study cohort, are at statistically increased risk of osteoporotic fractures.^48^^,^^49^ This may have further compounded their likelihood of developing VCFs following SBRT. Breast cancer histology serving as a potential confounder to female sex predicting VCF development was tested but demonstrated no statistical significance. Pre-existing VCFs at SBRT have previously been associated with subsequent VCF development following SBRT, perhaps owing to the already compromised integrity of the vertebral body.^21^^,^^22^^,^^30^^,^^44^^,^^45^ Importantly, this investigation found that pre-existing VCFs predicted progressive VCF development. The finding of lumbar region treatments associated with VCF development is also consistent with the literature on VCFs following SBRT.^21^^,^^27^^,^^28^ Most compression fractures, ranging from 60% to 75%, occur in the thoracolumbar region from T12 to L2 because of the transition from the more rigid thoracic spine to the relatively mobile lumbar spine.^49^ Greater SINS at SBRT has also previously been reported as individual predictors of VCF development following SBRT.^28^^,^^32^

Although lytic bone lesion types were not identified as a significant predictor of any VCF development following SBRT, lytic lesions were significantly predictive of de novo VCF development in this study. Lytic bone lesions have previously been associated with VCF development after SBRT, presumably because of their ability to weaken the bone prior to SBRT.^21^^,^^23^^,^^24^^,^^27^ Further, this study did not find margin dose, BED_10_, or prior irradiation history to significantly predict VCF development following SBRT. Although prior surgical history was not evaluated in this investigation, prior reports from our institution and others have indicated that a combination of kyphoplasty followed by adjuvant SBRT is safe and does not significantly increase the risk of VCF development.^40^^,^^50^^,^^51^

### Management of VCFs following SBRT

The choice of intervention following VCF after SBRT is determined by several factors, including the available treatment options at the institution, surgeon preference, and the patient's functional status, medical comorbidities, and systemic disease burden.^24^ Historically, the treatment of symptomatic VCFs has involved aggressive open stabilization with a combination of cement augmentation, pedicle screw reconstruction, and instrumented fusion.^52^ However, for fractures without significant kyphosis or substantial posterior element involvement, conservative management or percutaneous cement augmentation may offer an effective alternative with less morbidity compared with open surgical approaches.^52^ Less invasive procedures may help patients better tolerate and benefit from subsequent systemic oncologic therapies.

This study found that nearly half of patients with SBRT-induced VCFs were not clinically symptomatic enough to require surgical intervention. This may also be an underestimate, given limited survival rates and the lack of routine screening to detect asymptomatic VCFs. For patients who did ultimately require surgical intervention, less invasive palliative surgical procedures were generally preferred. In this study, 58% of VCFs were managed with subsequent stabilization surgery; rates from prior reports range from 25% to 60%.^21^, ^22^, ^23^, ^24^^,^^26^^,^^28^^,^^30^^,^^32^^,^^44^^,^^45^ Few studies have reported on outcomes for symptomatic VCFs following SBRT; none have explicitly reported on risk factors.^26^^,^^31^ In the present study, treatments in the lumbar spine and those with greater SINS scores and pre-existing VCFs were at greater risks of developing VCFs requiring subsequent spine surgery.

### Limitations

This study is a single-institution retrospective analysis with limitations arising from its design. The cohort included patients with varying degrees of neurologic and physical function, different primary tumor histology and systemic disease, and spinal stability. Additionally, this study encompasses advancements in SBRT technology and evolving radiation prescription regimens and SBRT treatment-related dosimetry metrics. Detailed volumetric dosimetry variables that have been previously associated with VCF risk such as planning target volume, vertebral body dose-volume parameters, and irradiated bone volume were not consistently available across the full study period because of changes in treatment planning platforms and electronic health records over 2 decades. The absence of these data limits our ability to evaluate potential dose-volume-fracture relationships, including whether larger planning target volumes or greater high-dose exposure to structurally critical portions of the vertebral body independently contribute to post-SBRT VCF development. Future prospective multi-institutional studies should incorporate standardized vertebral dose-volume metrics to improve risk stratification, more comprehensively evaluate dosimetry predictors, and facilitate development of dosimetry-informed VCF prediction models. Furthermore, targeted therapies, immunotherapies, and chemotherapy regimens have all continued to improve, contributing to prolonged patient survival following SBRT. The study did not account for the timing of these therapies in relation to SBRT. Likewise, use of bone-modifying osteoporotic agents may influence fracture risk and could confound VCF development associations. However, osteoporotic medication exposure (type, dose, duration, and timing relative to SBRT) was not consistently documented across the full retrospective study period and could not be reliably ascertained for all patients, including those treated outside our health system. Future prospective studies should systematically capture osteoporotic agent use to better define its relationship with VCF risk following spine SBRT. Long-term multicenter prospective studies are needed to fully delineate predictive risk factors of VCF development, timing, and severity following spine SBRT.

## Conclusions

VCFs following SBRT for spinal metastatic disease may be a debilitating complication, frequently leading to significant pain and neurologic deficits. In one of the largest studies to date, we report results from a large single-institution analysis on predictive risk factors of VCF development in specific patient subgroups following SBRT for vertebral metastases. Using 2 independent statistical models, we identified female sex, lumbar region treatments, and pre-existing VCF at SBRT as significant predictors of any VCF development. Osteolytic disease and epidural tumor extension predicted de novo VCFs, whereas pre-existing VCFs at SBRT predicted progressive VCFs following SBRT. Lumbar lesions, pre-existing VCFs at SBRT, and a greater SINS at SBRT were associated with VCFs requiring subsequent stabilization. Collectively, these findings highlight specific patient populations that may benefit from more frequent follow-up and consideration of individualized discussions regarding potential conservative or stabilization management approaches, particularly for those patients with multiple underlying risk factors. Together with previous studies, this research will help inform personalized clinical decision-making and enhance care for individualized at-risk patient populations.

## Disclosures

The authors declare that they have no known competing financial interests or personal relationships that could have appeared to influence the work reported in this paper.

## Appendix Supplementary materials

VCF Supplementary Tables 11 22 2025 Image, application 1

## Footnotes

Supplementary material associated with this article can be found in the online version at doi:10.1016/j.adro.2026.102033.
