# Supplementary material for: Risk Factors for Vertebral Compression Fracture Following Spine Stereotactic Body Radiation Therapy
Source: Adv Radiat Oncol. 2026 Mar 27;11(6):102033. doi: 10.1016/j.adro.2026.102033 (PMC13140028; doi:10.1016/j.adro.2026.102033)
Supplement: VCF Supplementary Tables 11 22 2025 [file mmc1.docx]

**Supplementary Table 1: Baseline patient demographics and clinical characteristics**

| **Characteristics** | **Value** |
| --- | --- |
| Total no. of pts. | 404 |
| Male | 210 (52%) |
| Female | 194 (48%) |
| Age at SBRT (yrs.) | 60 [22 – 94] |
| KPS at SBRT | 80 [50 – 100] |
| Total no. of treatments | 600 |
| Total no. of vertebral bodies | 779 |
| Primary tumor histology (no.) |  |
| Breast | 134 (22%) |
| Lung | 113 (19%) |
| Renal | 97 (16%) |
| Gastrointestinal | 73 (12%) |
| Melanoma | 38 (6%) |
| Prostate | 32 (5%) |
| Thyroid | 26 (4%) |
| Other | 87 (15%) |
| EBRT prior to SBRT (no.) | 397 (66%) |
| Chemotherapy at or prior to SBRT (no.) | 421 (70%) |
| Degree of systemic disease (no.) |  |
| Solitary (1 distant metastasis to the spine) | 108 (18%) |
| Oligometastatic (2-5 distant metastases) | 332 (55%) |
| Polymetastatic (>5 distant metastases) | 123 (21%) |
| Unknown | 37 (6%) |

No. = number. Pts. = patients. Yrs. = years. KPS = Karnofsky Performance Score. SBRT = stereotactic body radiation therapy. Values are reported as number (percentage) or median [range]. All % are divided by the total number of SBRT treatments unless otherwise stated.

**Supplementary Table 2: Tumor and spine SBRT treatment characteristics**

| **Characteristics** | **Value** |
| --- | --- |
| Total no. of treatments | 600 |
| Total no. of vertebral bodies | 779 |
| Tumor Location in the spine (no.) |  |
| Cervical | 81 (14%) |
| Thoracic | 266 (44%) |
| Lumbar | 164 (27%) |
| Sacral | 89 (15%) |
| ESCC at SBRT (no.) |  |
| 0 | 296 (49%) |
| 1a | 92 (15%) |
| 1b | 82 (14%) |
| 1c | 54 (9%) |
| 2 | 58 (10%) |
| 3 | 18 (3%) |
| Paraspinal musculature extension of tumor at SBRT (no.) | 215 (36%) |
| Bone lesion at SBRT (no.) |  |
| Lytic | 359 (60%) |
| Blastic | 92 (15%) |
| Mixed | 149 (25%) |
| Radiographic spinal misalignment at SBRT (no.) | 99 (17%) |
| Baseline vertebral body collapse at SBRT (no.) | 180 (30%) |
| Posterolateral spine involvement at SBRT (no.) | 370 (62%) |
| SINS at SBRT (no.) |  |
| 0-6 | 249 (42%) |
| 7-12 | 303 (51%) |
| 13-18 | 48 (8%) |
| Single-fraction SBRT (no.) | 493 (82%) |
| Margin dose (Gy) | 16 [8 – 20] |
| Maximum dose (Gy) | 20 [13 – 32] |
| Tumor volume (cc) | 30.4 [0.1 – 232.7] |
| Maximum dose to the spinal cord or cauda equina (Gy) | 10 [2 – 17] |
| Multi-fraction SBRT (no.) | 107 (18%) |
| Margin dose (Gy) | 24 [18 – 35] |
| Maximum dose (Gy) | 45 [29 – 60] |
| Tumor volume (cc) | 32.1 [2.1 – 264.3] |
| Maximum dose to the spinal cord or cauda equina (Gy) | 15 [8 – 46] |
| BED_2_ prescription dose (Gy) | 144 [40 – 220] |
| BED_10_ prescription dose (Gy) | 42 [14 – 60] |
| Isodose (%) | 80 [37 – 100] |

No. = number. SBRT = stereotactic body radiation therapy. ESCC = Epidural Spinal Cord Compression scale. SINS = Spinal Instability Neoplastic Score. GTV = gross tumor volume. BED_2_ = biologically equivalent dose with α/β = 2 Gy. BED_10_ = biologically equivalent dose with α/β = 10 Gy. Values are reported as number (percentage) or median [range]. All % are divided by the total number of SBRT treatments unless otherwise stated.

| **Supplementary Table 3: A comparison of published studies on prognostic risk factors for VCF development following SBRT** | | | | | | | | |
| --- | --- | --- | --- | --- | --- | --- | --- | --- |
| **Study** | **No. of patients (no. of segments)** | **Follow-up (months)** | **% Post-SBRT VCF development rate (de novo/ progressed)** | **Time to VCF development (months)** | | **% VCFs requiring post-SBRT stabilization** | **Risk factors on UVA** | **Risk factors on MVA** |
| Rose et al. (2009)^27^ | 62 (71) | 13 (N/A) | 39 (N/A) | 1. (N/A) | 25 | | None reported | Lytic lesion  Lesions occupying 41%-60% of VB  Thoracolumbar/lumbar location |
| Boehling et al. (2012)^21^ | 93 (123) | 15 (1-71) | 20 (11/9) | 3 (N/A) | 60 | | Age > 55  Pre-existing VCF  Baseline pain and narcotic use  Post-SBRT pain and narcotic use | Age > 55  Pre-existing VCF  Lytic lesion |
| Cunha et al. (2012)^23^ | 90 (167) | 7.4 (0.4-37.3) | 11.4 (7.2/4.2) | 2 (0.5-21.6) | 47 | | Kyphosis/scoliosis  Lytic lesion  Prior VCF  Histology  Dose/fx ≥ 20 Gy | HCC histology  Lytic lesion  Kyphosis/scoliosis  Dose/fx ≥ 20 Gy  Lung histology |
| Sahgal et al. (2013)^24^ | 252 (410) | 11.5 (0.03-113.2) | 13.9 (6.6/7.3) | 2.5 (0.03-43.01) | 43 | | Dose/fx  VB collapse  > 50% VB involved by tumor  Lytic lesion  Spinal misalignment  Paraspinal/epidural extension | Dose/fx  Pre-existing VCF: < 50% and > 50%  > 50% VB involved by Tumor  Lytic lesion  Spinal misalignment |
| Sung et al. (2014)^25^ | 72 (72) | 11* (3-24) | 36 (N/A) | 1.5* (0.3-3.5) | 58 | | Pre-SBRT deformity  VB osteolysis rate  SINS Score  Whole VB involvement | VB osteolysis rate ≤ 60% vs > 60% |
| Jawad et al. (2016)^22^ | N/A (594) | 10.1 (0.03-57) | 5.7 (3/2.7) | 3 (N/A) | N/A | | Short interval from diagnosis to SBRT  Solitary metastasis  No additional bony metastases  No prior chemotherapy  Pre-existing VCF  No MRI for target delineation  EQD_2_ tumor ≥ 41.8 Gy  Max dose to spinal cord > 46.1 Gy  EQD_2_ tumor volume > 37.3 cm | Solitary metastasis  Pre-existing VCF  Prescription dose to target volume dose ≥ 38.4 Gy EQD_2_ |
| Germano et al. (2016)^42^ | 79 (143) | 16* (3-78) | 21 (6.3/14.7) | 5* (3-24) | 30 | | Histology  SINS  VB collapse  Pre-existing VCF  Baseline pain | None reported |
| Lee et al. (2016)^32^ | 79 (100) | 21.2 (N/A) | 32 (20/12) | 3.3 (0.4-34.1) | 47 | | SINS Score  ESCC classification  Dose/fraction < 20 Gy vs 24 Gy  Age < 65 vs ≥ 65  Prior XRT failure | SINS 0-6 vs 7-12  Age < 65 vs ≥ 65 |
| Thibault et al. (2016)^43^ | 55 (100) | 7.3 (0.6-67.6) | 17 (5/12) | 1.68 (0.76-7.79) | N/A | | Pre-existing VCF  Lytic lesion  Dose/fx ≥ 20 Gy | ≥11.6% lytic lesion  Pre-existing VCF  Dose/fx ≥ 20 Gy |
| Virk et al. (2017)^26^ | 323 (552) | 12.6 (3.7-31.9) | 8.2 (5.1/3.1) | 13.2 (6.3-28.7) | 58 | | None reported | None reported |
| Boyce-Fappiano et al. (2017)^30^ | 448 (1070) | 17.7 (N/A) | 11.9 (4.2/4.1) | 2.7 (0.16-54.9) | 29 | | 3+ treated levels  Lytic lesion  Hematologic malignancies  Pre-existing VCF  Female sex  Thoracic spine | 3+ treated levels  Lytic lesion  Pre-existing VCF |
| Kowalchuk et al. (2022)^28^ | 331 (464) | 21 (11-39) | 18 (14/4) | 9 (3-21) | N/A | | None reported | SINS Score > 6  GTV > 10 cc  Lumbar location  Epidural tumor extension |
| Current study | 404 (779) | 8 (1-251) | 10 (5/5) | 6 (1-83) | 58 | | Female sex  Lumbar lesion  >1 VB irradiated per treatment  Pre-existing VCF  SINS Score > 6 | Female sex  Pre-existing VCF  Lumbar location |
| VCF = vertebral compression fracture. SBRT = stereotactic body radiation therapy. UVA = univariable analysis. MVA = multivariable analysis. VB = vertebral body. SBRT = stereotactic body radiation therapy. GTV = gross tumor volume. SINS = spinal instability neoplastic score. Fx = fractions. ESCC = epidural spinal cord compression. EQD_2_ = equivalent dose in 2 fractions. * = mean. Follow-up and time to VCF development are reported as median (range) unless otherwise stated. Studies with > 50 patients evaluating risk factors for VCFs following SBRT were included in Supplementary Table 3. | | | | | | | | |
